# The dominant-negative interplay between p53, p63 and p73: A family affair

**DOI:** 10.18632/oncotarget.11774

**Published:** 2016-08-31

**Authors:** Olivier Billant, Alice Léon, Solenn Le Guellec, Gaëlle Friocourt, Marc Blondel, Cécile Voisset

**Affiliations:** ^1^ Inserm UMR 1078, Université de Bretagne Occidentale, Faculté de Médecine et des Sciences de la Santé, Etablissement Français du Sang (EFS) Bretagne, CHRU Brest, Hôpital Morvan, Laboratoire de Génétique Moléculaire, Brest, France

**Keywords:** p53, p63, p73, dominant-negative effect, yeast

## Abstract

The tumor suppression activity of p53 is frequently impaired in cancers even when a wild-type copy of the gene is still present, suggesting that a dominant-negative effect is exerted by some of p53 mutants and isoforms. p63 and p73, which are related to p53, have also been reported to be subjected to a similar loss of function, suggesting that a dominant-negative interplay might happen between p53, p63 and p73. However, to which extent p53 hotspot mutants and isoforms of p53, p63 and p73 are able to interfere with the tumor suppressive activity of their siblings as well as the underlying mechanisms remain undeciphered. Using yeast, we showed that a dominant-negative effect is widely spread within the p53/p63/p73 family as all p53 loss-of-function hotspot mutants and several of the isoforms of p53 and p73 tested exhibit a dominant-negative potential. In addition, we found that this dominant-negative effect over p53 wild-type is based on tetramer poisoning through the formation of inactive hetero-tetramers and does not rely on a prion-like mechanism contrary to what has been previously suggested. We also showed that mutant p53-R175H gains the ability to inhibit p63 and p73 activity by a mechanism that is only partially based on tetramerization.

## INTRODUCTION

The transcription factor p53 is a key tumor suppressor referred to as the “guardian of the genome” [1]. In response to a broad range of cellular stresses [2] p53 elicits genome maintenance by inducing cell cycle arrest and DNA repair or leads cells toward apoptosis when they are irreversibly damaged [3, 4]. It is no surprise then that the inactivation of p53 by mutation is the most common genetic alteration in human tumors [5–7].

**Figure 1:**
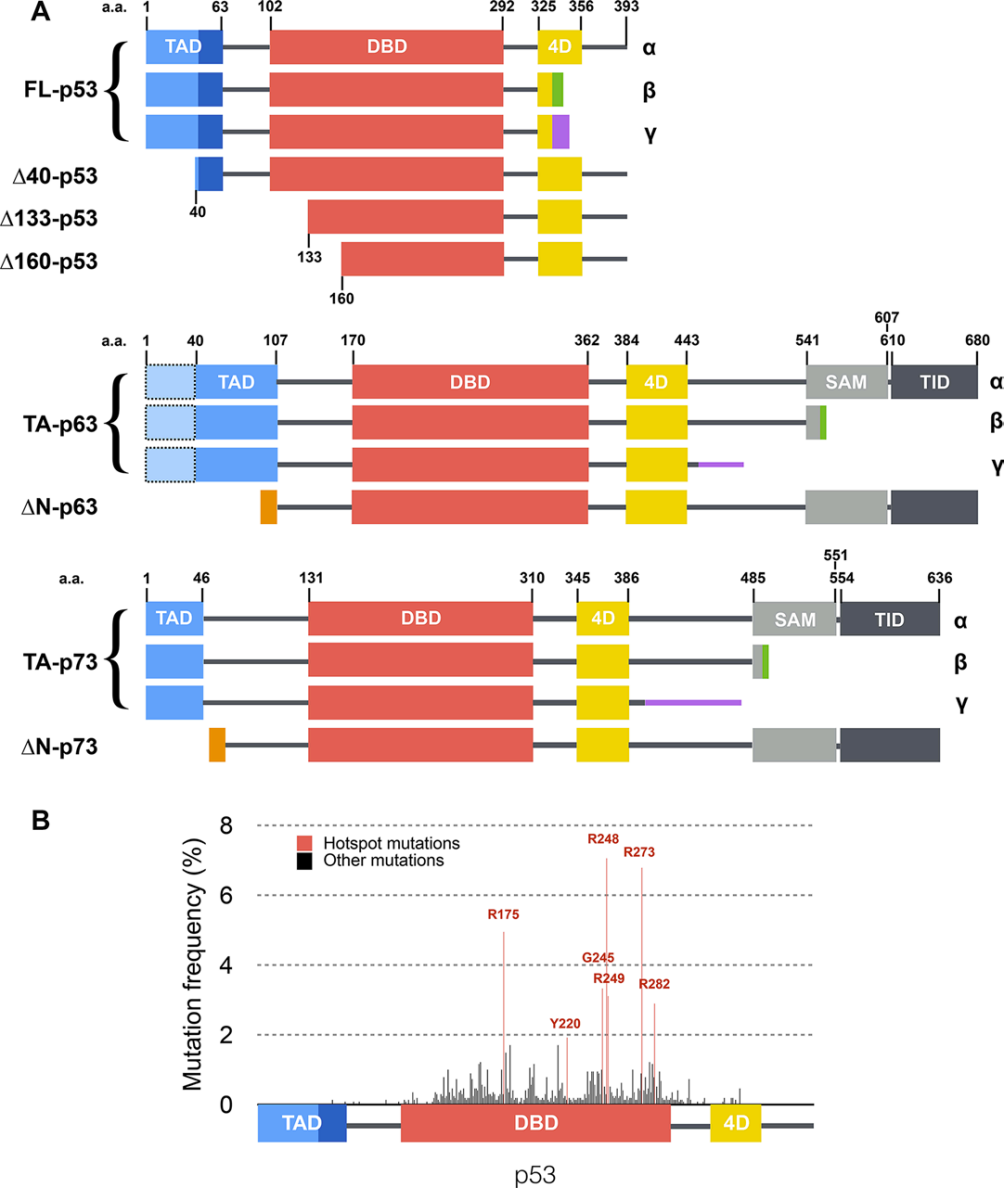
Structure of p53 family isoforms and distribution of mutations within p53 (**A**) Main transcripts of p53 family. Isoforms are generated through secondary initiation codons (Δ40, Δ133 or Δ160) or alternate splicing sites (α, β, γ… C-termini) leading to multiple combinations. p63 and p73 present several other N-terminal and C-terminal isoforms [19] that are not depicted here. p53, p63 and p73 share a similar modular organization with one or two transcription activation domains (TAD), a DNA binding domain (DBD), a tetramerization domain (4D) and two domains specific to p63 and p73: a sterile alpha motif (SAM) and a transcription inhibition domain (TID). (**B**) Distribution and frequency of p53 mutations described in human tumors (data from the International Agency for Research on Cancer). Hotspot mutations are shown in red and are all located within the DNA binding domain of p53.

p53 was long thought to be one of its kind until the identification of p63 [8] and p73 [9, 10] which present similar modular structures and a high degree of homology with p53 [11, 12]. Both p63 and p73 share with p53 common target genes involved in apoptosis and cell cycle arrest, which makes them potent tumor suppressor genes [13] although it is not their primary function [14–18]. *TP53*, *TP63* and *TP73* encode a wide range of isoforms combining shorter or alternative N-terminal extremities (TA, FL, and ΔN isoforms) with alternative C-terminal sequences (α, β and γ isoforms; Figure 1A) [19]. Their role in cell fate determination is finely orchestrated through tissue specific localization [20, 21], expression level [22–24] and time-dependent regulation [25].

In contrast with most tumor suppressor genes whose expression is lost during tumorigenesis, p53 remains expressed though frequently affected by missense mutations. Seven mutational hotspots have been identified (Figure 1B) that are strongly associated with poor prognosis for patients [26–28]. Certain mutations of p53 have been described as dominant-negative [29–32] as they lead to a loss of the tumor-suppressive function of the remaining wild-type p53 allele. Moreover, some p53 mutants have also been shown to exert a dominant-negative effect over the related proteins p63 and p73, which virtually leads to a complete shutdown of p53 family activity [33–38]. Several explanations have been provided regarding the ability of p53 dominant-negative mutants to disrupt WT (wild-type) p53 function. Whether it happens through (i) a competitive effect where mutants or isoforms starve wild-type protein from binding sites or co-factors [39], (ii) the formation of inactive hetero-oligomers containing both WT and mutant p53 proteins [39, 40], or (iii) a prion-like mechanism by which a mutant p53 drives the WT protein into an alternate inactive conformation [41–43] remains discussed to date.

Although p63 and p73 are scarcely found mutated in cancers [44–48], the expression pattern of their isoforms is severely deregulated in cancers. The ratio of expression levels between the different isoforms seems indeed critical for their tumor-suppressive function [20, 33]. Whereas TA/FL isoforms are generally considered as tumor suppressors [37, 49, 50], their ΔN counterparts that are crucial to epidermis development (ΔN-p63, [12]) or neural development (ΔN-p73, [18]) have been shown to be oncogenic factors involved in the dominant-negative effect by some studies [20, 25, 51] but have also been shown to be transcriptionally active by others [52–55]. Overall, the progressive identification of said isoforms has revealed a whole new level of complexity of p53 family but their exact role in carcinogenesis remains undeciphered [24, 56, 57].

Among the models used to study p53 family members, yeast has received much attention as it can be used to evaluate human p53 transcriptional activity using FASAY (Functional Analysis of Separated Alleles of p53 in Yeast) [58–60]. FASAY is based on engineered yeast strains whose genome contains a reporter gene which expression is driven by a specific p53 response element. In addition, yeast is a naive eukaryotic system regarding p53 as it lacks a p53 orthologue. Hence, FASAY has allowed the identification of tumor-derived loss-of-function mutations of p53 [61, 62] and later the characterization of dominant-negative mutations of p53 [29, 30, 63]. In addition, due to the strong homology between p53, p63 and p73 DNA binding domains [64], FASAY has been successfully used to evaluate p63 and p73 transactivity [54].

In this study, we investigated the mechanisms of the dominant-negative effect of 7 hotspot p53 mutants and of the 24 main isoforms of p53, p63 and p73 using FASAY strains. We show that only mutants and isoforms of p53 that are both inactive and able to form tetramers can impair the transcriptional activity of p53-WT. Indeed, we showed that their dominant-negative effect relies on tetramer-poisoning. We found no evidence that a prion-based mechanism is involved in the dominant-negative behavior of the p53 and p73 proteins tested. Finally, we report that mutant p53-R175H gains the ability to interact with p63 and p73 and that this interaction is only partially based on tetramerization.

## RESULTS

### Loss-of-function hotspot mutants and isoforms of p53 impair FL-p53α-WT transcriptional activity

**Figure 2:**
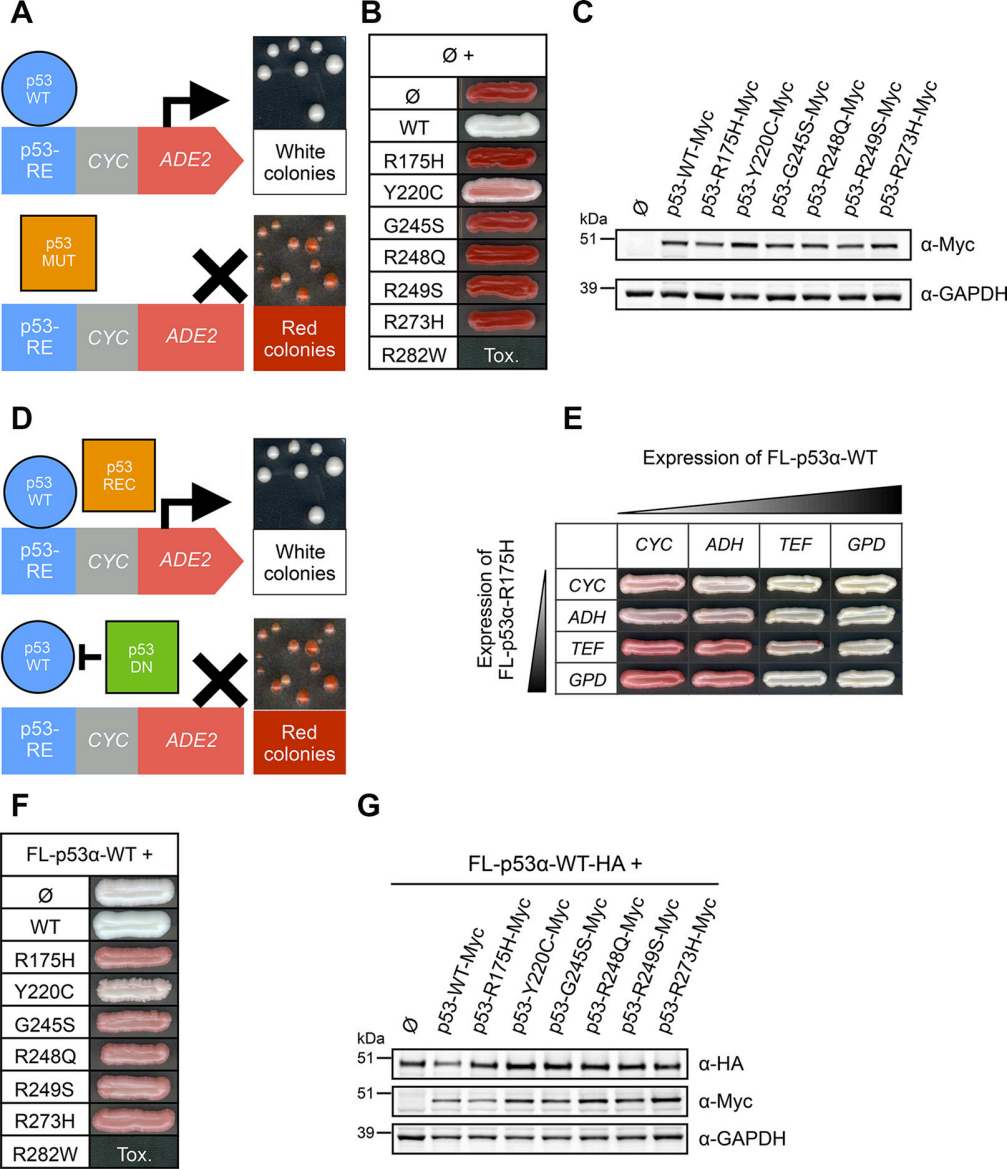
Transcriptional activity and dominant-negative effect of hotspot mutants of p53 in FASAY-RGC strain (**A**) Principle of FASAY. FASAY is based on the use of the yeast *ADE2* reporter gene, which encodes AIR carboxylase (Ade2p), an enzyme involved in the adenine biosynthesis pathway. The *ADE2* gene is placed under the control of the p53 response element (RE) of *p21* or *RGC* genes fused to the weak yeast *CYC1* promoter deprived of upstream activator sequences (UAS). Owing to the absence of Ade2p enzyme, its substrate phosphoribosylaminoimidazole (AIR) accumulates and once oxidized by respiration gives a red coloration to yeast colonies. When expressed, functional p53 (blue circle) binds to p53-RE and induces the production of a quantity of Ade2p sufficient to obtain white colonies. In contrast, in cells expressing a loss-of-function mutant of p53 (orange square), the expression of *ADE2* is not induced and the lack of Ade2p leads to red colonies. Any intermediate amount of Ade2p leads to pink colonies, whose color intensity is proportional to the transcriptional activity of the expressed protein. (**B**) Transcriptional activity of hotspot p53 mutants. All hotspot mutants of p53 were expressed under the control of the strong *GPD* promoter. Tox indicates the absence of cell growth due to an excessive level of expression of the mutant. (**C**) Expression level of Myc-tagged p53 mutants was analyzed by western blotting using anti-Myc antibodies. GAPDH was used as a loading control. (**D**) Principle of the transdominance assay. A transactive FL-p53α-WT protein (blue circle) was co-expressed with an inactive mutant of p53. A recessive mutant of p53 (orange square) would not interfere with the transactivity of FL-p53α-WT and could thus lead to white colonies. A dominant-negative mutant of p53 (green square) would prevent WT p53 activity and would thus lead to the formation of red colonies. Intermediate color phenotypes indicate a partial dominant-negative effect of the mutant. (**E**) Dominant-negative effect of mutant FL-p53α-R175H over FL-p53α-WT in FASAY-RGC. FL-p53α-WT and mutant FL-p53α-R175H were co-expressed under the control of promoters of increasing strength (*CYC*<*ADH*<*TEF*<*GPD*). (**F**) Transdominance assay of hotspot mutants of p53 over FL-p53α-WT. Mutants were expressed under the control of the strong *GPD* promoter and FL-p53α-WT was expressed under the control of the moderate *ADH* promoter. (**G**) Western blot analysis of the expression level of WT FL-p53α-HA and mutant FL-p53α-Myc using anti-HA and anti-Myc antibodies. GAPDH was used as a loading control.

_I_n order to identify loss-of-function p53 hotspot mutants that are able to interfere with FL-p53α-WT function, we first tested the transcriptional activity of seven hotspot FL-p53α mutants using FASAY, which principle is presented in Figure 2A. p53 mutants were expressed in FASAY strains containing RGC (FASAY-RGC, Figure 2B) or p21 (FASAY-p21, Supplementary Figure S1A) response elements (RE). As expected, FL-p53α-WT positive control led to white colonies. We observed that all hotspot mutants but Y220C induced the formation of red colonies in both FASAY strains despite all being expressed at levels similar to FL-p53α-WT (Figure 2C and Supplementary Figure S1B), which indicates their inability to induce transcription in yeast [65]. R282W strongly inhibited cell growth at high expression level (Figure 2B) as previously described [66] but remained inactive at a lower expression level (Supplementary Figure S1C). Next we took advantage of FASAY to evaluate the dominant-negative potential of p53 hotspot mutants: a dominant-negative mutant would induce a red phenotype by preventing the activity of the co-expressed FL-p53α-WT (Figure 2D). We first evaluated the dominance of R175H over p53-WT. In line with previous reports, R175H exhibited a dominant effect with the intensity of the color phenotype depending on R175H/p53-WT expression ratio (Figure 2E): a light pink coloration of colonies expressing R175H at a level similar than p53-WT indicates a dominant-negative effect which is in good agreement with previous reports [29, 30, 54, 63]. The color shift was stronger when R175H was expressed at a higher level than p53-WT and was proportional to the level of expression of p53-R175H (Figure 2E). As we needed a pronounced readout to distinctly visualize the dominant-negative effect of mutants and isoforms, we expressed p53 mutants and isoforms under the control of the strong *GPD* promoter and wild-type p53 under the control of the moderate *ADH* promoter. Of note, the difference in steady state level we obtained between mutant and WT p53 is rather limited (2 to 3 fold) and is physiologically relevant since p53 mutants frequently accumulate in cancer cells [67, 68] and ΔN isoforms of p73 are found overexpressed in various cancer cell types [13]. Co-expression of FL-p53α-WT with itself and with Y220C which is significantly active served as controls and both led to white colonies. All loss-of-function hotspot mutants of p53 induced the formation of dark pink colonies using FASAY-RGC (Figure 2F) and FASAY-p21 (Supplementary Figure S1D) despite being all expressed at a level similar to FL-p53α-WT and Y220C (Figure 2G and Supplementary Figure S2E), confirming previous reports [29, 32, 63]. Of note, R282W remained toxic for yeast when co-expressed under the *GPD* promoter with FL-p53α-WT. We thus evaluated the dominant-negative potential of R282W by placing it under the control of the same promoter than FL-p53α-WT (*ADH*) and found that it was not able to interfere with p53-WT transcriptional activity in such conditions (Supplementary Figure S1F). Hence, all loss-of-function hotspot mutants tested but R282W displayed a dominant-negative effect over FL-p53α-WT.

**Figure 3:**
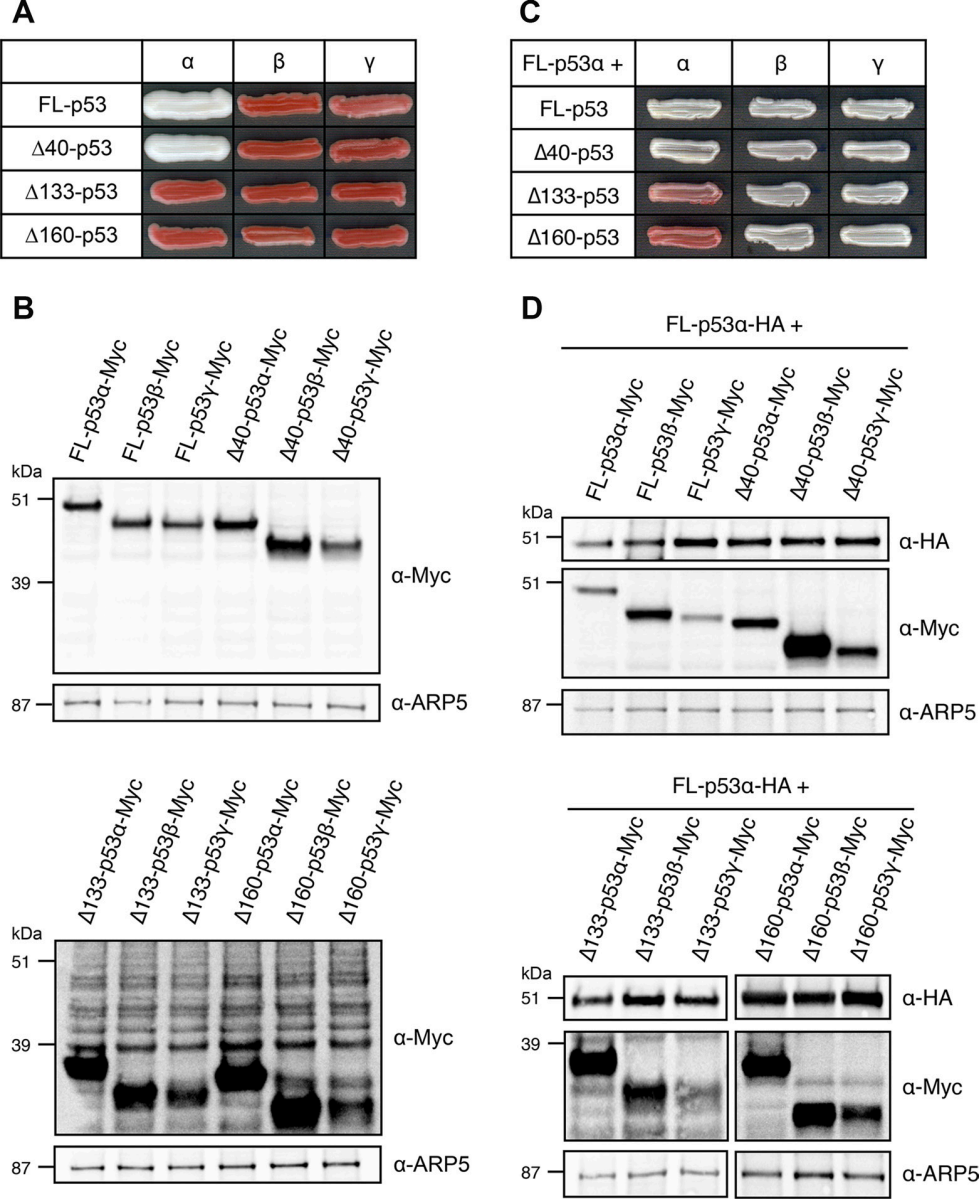
Transcriptional activity and dominant-negative effect of p53 main isoforms in FASAY-RGC strain (**A**) Transcriptional activity of p53 main isoforms expressed under the control of the strong *GPD* promoter. (**B**) Western blot analysis of the expression level of Myc-tagged p53 isoforms using anti-Myc antibodies. ARP5 was used as a loading control. (**C**) Dominant-negative effect of FL-, Δ40-, Δ133- and Δ160-p53 isoforms over FL-p53α-WT. Isoforms of p53 were expressed under the control of the strong *GPD* promoter and FL-p53α-WT-HA was expressed under the control of the mild *ADH* promoter. (**D**) Western blot analysis of the expression level of FL-p53α-WT-HA and Myc-tagged isoforms FL-, Δ40-, Δ133- and Δ160-p53 using anti-HA and anti-Myc antibodies. ARP5 was used as a loading control.

We then evaluated the dominant-negative potential of 12 of the most common p53 isoforms. We first identified transcriptionally active isoforms of p53 using FASAY-RGC (Figure 3A) and FASAY-p21 strains (Supplementary Figure S1G): FL-p53α and Δ40-p53α were functional transcription factors whereas all the other isoforms were inactive despite being all expressed at similar levels (Figure 3B). Δ133-p53α and Δ160-p53α were transcriptionally inactive, which was expected since they have no TAD and a truncated DBD (Figure 1A). Of note, FL-p53β/γ and Δ40-p53β/γ were also inactive in yeast while presenting complete or partial TADs. Among the ten p53 isoforms found to be inactive, only Δ133-p53α and Δ160-p53α exerted a dominant-negative effect over FL-p53α-WT in FASAY-RGC (Figure 3C) and in FASAY-p21 (Supplementary Figure S1H) despite being expressed at similar levels than most of the other inactive isoforms (Figure 3D). Δ133- and Δ160-p53β/γ differ from their α counterparts by an alternate C-terminal end harboring an altered tetramerization domain (Figure 1A). However, Δ133-p53γ and Δ160-p53γ expression levels being low, we cannot exclude that these isoforms may exert a dominant-negative effect when expressed at a higher expression level. Taken together, our results strongly suggest that all the mutants and isoforms of p53 which are transcriptionally inactive and that retain an intact tetramerization domain are able to reduce FL-p53α-WT activity.

### The dominant-negative effect of p53-R175H mutant does not rely on prion properties in yeast

**Figure 4:**
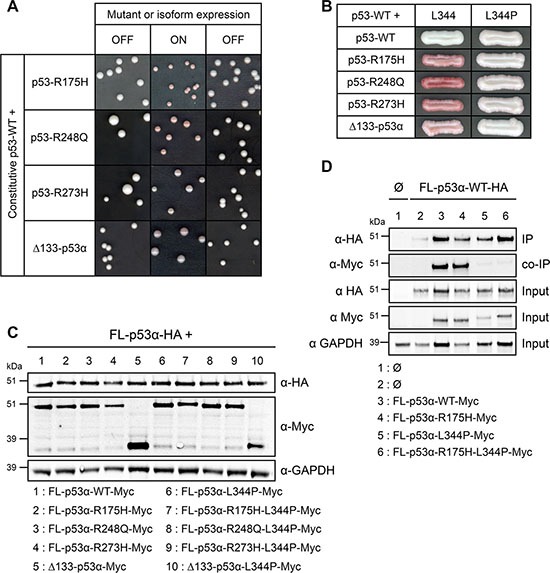
Mechanistic insights into the dominant-negative effect of mutants and isoform of p53 in FASAY-RGC strain (**A**) Prion propagation assay of the dominant-negative R175H, R248Q and R273H mutants and Δ133-p53α isoform in yeast. Wild-type FL-p53α expression was placed under the control of the *GPD* constitutive promoter whereas the expression of R175H mutant was put under the control of the glucose-repressible *GAL* promoter which is switched ON by galactose and switched OFF by glucose. (**B**) Impact of the tetramerization disruptive L344P mutation on dominant-negative mutants and on Δ133-p53α isoform of p53. R175H, R248Q and R273H mutants and Δ133-p53α isoform, as well as the double mutants R175H-L344P, R248Q-L344P and R273H-L344P and the Δ133-p53α-L344P mutant isoform placed under the control of the *GPD* strong promoter were co-expressed with FL-p53α-WT plad under the control of the moderate *ADH* promoter. (**C**) Western blot analysis of the expression level of HA-tagged FL-p53α-WT and Myc-tagged dominant-negative mutants R175H, R248Q and R273H and isoform Δ133-p53α harboring the L344P mutation using anti-HA and anti-Myc antibodies. GAPDH was used as a loading control. (**D**) L344P mutation impedes p53 tetramerization. FL-p53α-WT-HA was co-expressed along with FL-p53α-WT-Myc, FL-p53α-R175H-Myc, FL-p53α-L344P-Myc or FL-p53α-R175H-L344P-Myc. FL-p53α-WT-HA was immunoprecipitated using a rat anti-HA antibody. The immune complexes were subjected to western blotting. Immunoprecipitated HA-tagged proteins (IP) and co-immunoprecipitated Myc-tagged proteins (Co-IP) were detected using rabbit anti-HA and mouse anti-Myc antibodies, respectively. 25 μg of the extract used for the immunoprecipitations were loaded as a control for the expression of HA- and Myc-tagged proteins (Input).

The mechanism behind the dominant-negative effect of p53 remains discussed to date. Recent works support a prion-like-based dominant-negative effect for some hotspot mutants of p53 [43]. Prions are infectious proteins able to form amyloid aggregates that self-propagate by an autocatalytic folding process [69]. Prion traits are thereby dominant and are transmitted over cell division. Prions exist in yeast [70, 71] and at least some of the mechanisms controlling prions appearance and maintenance are conserved from yeast to mammals [72]. We thus examined an essential prion property: the autocatalytic propagation. To assess the potential propensity of R175H, R248Q, R273H and Δ133-p53α to transmit their dominant-negative features to p53-WT by a prion-based mechanism, these mutant proteins and this isoform were expressed under the control of a glucose-repressible GAL promoter, together with a constitutively expressed FL-p53α-WT: in the presence of galactose (ON condition), either R175H, R248Q, R273H or Δ133-p53α were co-expressed with FL-p53α-WT in FASAY-RGC (Figure 4A) and FASAY-p21 (Figure S2A) which systematically led to the formation of 100% of pink colonies due to the dominant-negative effect of R175H, R248Q, R273H and Δ133-p53α over FL-p53α-WT. However, when the expression of the dominant-negative mutants was repressed by addition of glucose, 100% of yeast colonies turned back to white. If R175H, R248Q, R273H and Δ133-p53α were prions, then colonies would have kept a pink phenotype despite the shut-off of their expression due to the transmission of the prion conformation to p53 WT proteins (Figure 4A and Supplementary Figure S2A). Hence, we concluded that at least in yeast, the dominant-negative effect of R175H, R248Q, R273H and Δ133-p53α over FL-p53α-WT does not rely on a prion-like mechanism.

### The dominant-negative effect of mutants and isoforms of p53 relies on their capacity to interact with p53-WT

Our results underline the crucial role of tetramerization in the dominant-negative effect of isoforms of p53, since it requires an intact tetramerization domain (Figure 3C). This led us to explore the hypothesis of a dominant-negative effect based on the formation of hetero-tetramers mixing transactive p53 with inactive dominant-negative isoforms or mutants [39, 73]. In this model, the potency of the dominant-negative effect should depend on the ratio between inactive and functional forms of p53. To challenge this hypothesis, we co-expressed increasing quantities of both FL-p53α-WT and the dominant-negative R175H using four different promoters of increasing strength. As shown in Figure 2E, the intensity of the red coloration was proportional to R175H expression level. Our results therefore indicate that R175H acts as a dose-dependent inhibitor of p53-WT which is in good agreement with our data showing that the dominant-negative effect of R175H, R248Q, R273H and Δ133-p53α is not based on a prion-like mechanism contrary to what has been previously suggested [43].

In order to test the potential role of hetero-tetramerization in the dominant-negative effect of p53 mutants and isoform, we introduced L344P mutation that has been described to prevent p53 tetramerization [74]. When co-expressed with FL-p53α-WT, dominant-negative mutants R175H, R248Q, R273H and isoform Δ133-p53α harboring the additional L344P mutation led to the formation of white colonies using FASAY-RGC (Figure 4B) and FASAY-p21 (Supplementary Figure S2B), despite being expressed at similar levels than the corresponding single hotspot mutants (Figure 4C), which indicates a complete loss of their dominant-negative potential. Co-immunoprecipitation showed a strong interaction of FL-p53α-WT with itself as well as with R175H and showed that L344P mutation abolished this interaction (Figure 4D). These data confirm that the dominant-negative effect exerted by loss-of-function mutants and isoform of p53 over FL-p53α-WT requires their ability to interact with the full-length protein through their tetramerization domain. Altogether, our data indicate that this dominant-negative effect is likely due to the formation of mixed tetramers that are less or not transcriptionally active anymore as a consequence of tetramer poisoning.

### Transcriptional activity of p63 and p73 main isoforms

**Figure 5:**
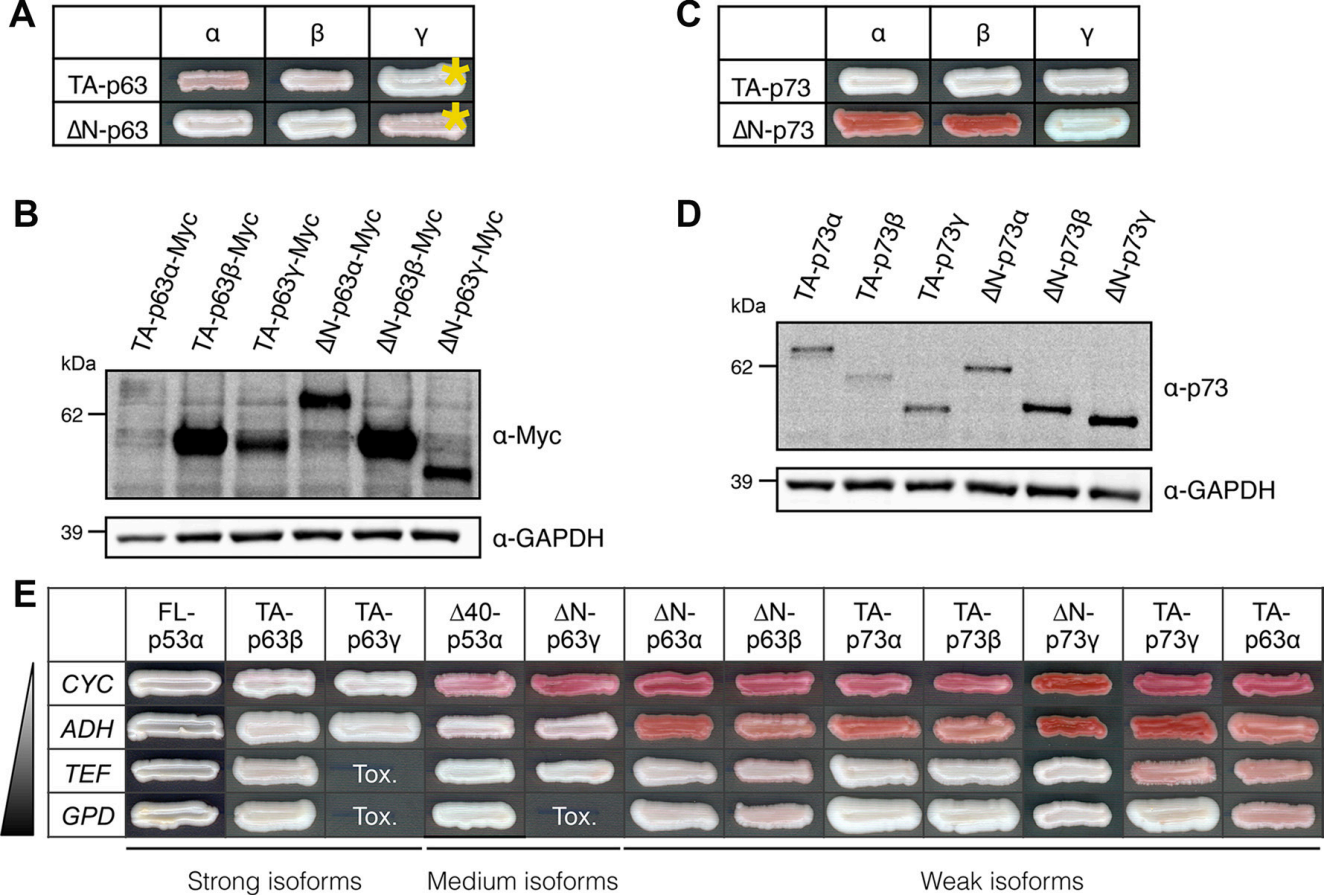
Transactivity of p63 and p73 isoforms in FASAY-RGC strain (**A**) Transcriptional activity of the 6 main isoforms of p63 expressed under the control of the strong *GPD* promoter. Yellow asterisks indicate isoforms that were expressed under the control of the moderate *ADH* promoter because of their toxicity when expressed from the *GPD* promoter. (**B**) Western blot analysis of the expression level of Myc-tagged p63 isoforms using anti-Myc antibodies. GAPDH was used as a loading control. (**C**) Transcriptional activity of the 6 main isoforms of p73 expressed under the control of the strong *GPD* promoter. (**D**) Western blot analysis of the expression level of p73 isoforms using anti-p73 antibodies. GAPDH was used as a loading control. (**E**) Transactive p53, p63 and p73 isoforms were expressed under the control of promoters of increasing strength (*CYC*<*ADH*<*TEF*<*GPD*). The most effective forms (“strong” isoforms) were FL-p53α, TA-p63γ and TA-p63γ as they allowed a color shift from red to white from the weakest promoter (*CYC*). Δ40-p53α and ΔN-p63γ were “medium” isoforms whereas all the other isoforms (“weak”) exhibited a significant activity only when expressed from stronger *TEF* and *GPD* promoters. Tox indicates the absence of cell growth due to an excessive level of expression of the isoform.

Although scarcely found mutated in cancers, p63 and p73 genes encode multiple isoforms, some of which have been reported to exert a dominant-negative effect [20, 25, 51]. We first used FASAY strains to identify isoforms of p63 and p73 that are transcriptionally active through binding on p53-specific *RGC* and *p21* response elements. In contrast to p53 isoforms, all TA- and ΔN-p63 isoforms tested were transcriptionally active in FASAY-RGC (Figure 5A) and FASAY-p21 (Supplementary Figure S2C). As previously described, the canonical isoform TA-p63α displayed the lowest transcriptional activity among p63 isoforms [75], which could be due to its weaker ability to form tetramers [54] or to its instability in yeast as suggested by its low expression level (Figure 5B). Among the 6 isoforms of p73 tested, TA-p73α, TA-p73β, TA-p73γ and ΔN-p73γ were transcriptionally active in FASAY-RGC (Figure 5C) and to a lesser extent in FASAY-p21 (Supplementary Figure S2C). In contrast, ΔN-p73α and ΔN-p73β were inactive in FASAY-RGC but weakly active in FASAY-p21 while being expressed at similar level than ΔN-p73γ (Figure 5D), which is consistent with a previous report [54].

We observed that TA-p63γ and ΔN-p63γ induced the inhibition of yeast growth when using a strong promoter but found them to be transcriptionally active at lower expression levels. We thus further investigated the transcriptional potential of each of the 12 transactive isoforms of p53, p63 and p73 by expressing them under the control of four promoters of different strength in the reporting system that we found to be the most sensitive (FASAY-RGC). This led us to classify p53, p63 and p73 isoforms in 3 classes of transactivity (referred as strong, medium and weak) based on the minimal level of expression required to trigger a detectable white phenotype in our assay (Figure 5E).

### Dominant-negative effect of p53-R175H mutant and different isoforms over p53, p63 and p73 isoforms

We next sought to identify potential dominant-negative interference of p53 mutant and isoforms on p53, p63 and p73 transcriptional activity. For this purpose, we co-expressed R175H, Δ133-p53α or Δ160-p53α together with each of the transactive isoforms of p53, p63 and p73 identified (Figures 3A, 5A, 5C): the intensity of the color shift induced by p53 mutant and isoforms compared to the negative control was a reflection of their dominant-negative potential. Our results that are summarized in Table 1 showed that inactive mutants and isoforms of p53 can interfere with active isoforms of p63 and p73 (Supplementary Figures S2D and S3).

**Table 1:** Dominant-negative effect of mutant p53-R175H and isoforms Δ133-p53α and Δ160-p53α over all transactive isoforms of the p53 family (Supplementary Figures S2D and S3)

	p53-R175H	Δ133-p53α	Δ160-p53α
**FL-p53α**	+++	+	+++
**Δ40-p53α**	++	+	+
**TA-p63α**	+	−	−
**TA-p63β**	+	−	−
**TA-p63γ**	+	−	−
**ΔN-p63α**	+	−	−
**ΔN-p63β**	++	−	++
**ΔN-p63γ**	+++	++	+++
**TA-p73α**	++	+	+
**TA-p73β**	++	−	+
**TA-p73γ**	+++	++	+++
**ΔN-p73γ**	+	−	−

**Figure 6:**
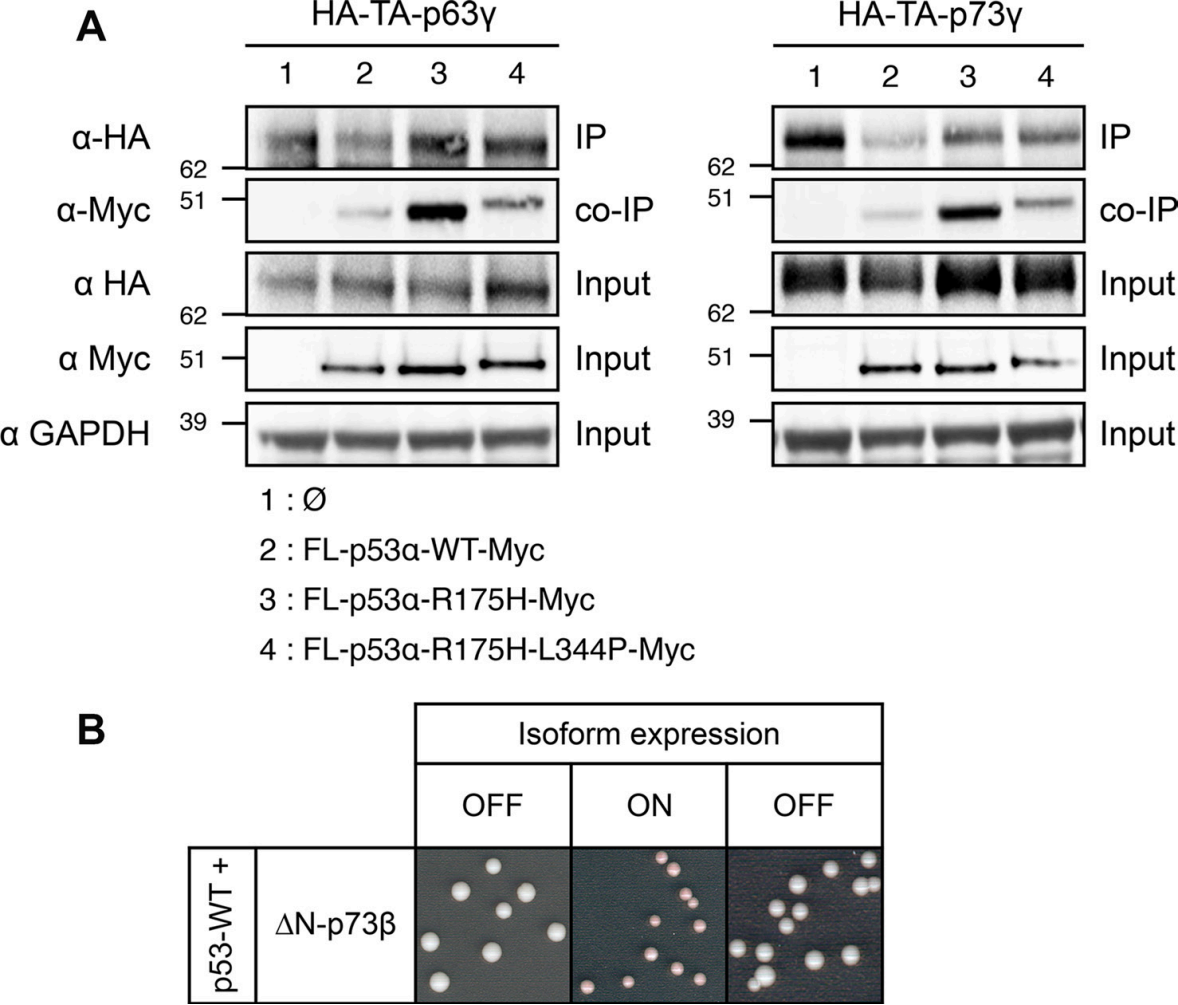
Investigation of the mechanism of the dominant-negative effect exerted by FL-p53α-R175H over TA-p63γ and TA-p73γ in FASAY-RGC strain (**A**) TA-p63γ-HA (left panel) or TA-p73γ-HA (right panel) was co-expressed with FL-p53α-WT-Myc, FL-p53α-R175H-Myc or FL-p53α-R175H-L344P-Myc. TA-p63γ-HA and TA-p73γ-HA proteins were immunoprecipitated using a rat anti-HA antibody (Roche). The immune complexes were subjected to western blotting. Immunoprecipitated HA-tagged proteins were detected using a rabbit anti-HA antibody (Clontech) and co-immunoprecipitated Myc-tagged proteins were detected using a mouse anti-Myc antibody (Clontech). 25 μg of the extract used for the immunoprecipitations were loaded as a control of the presence of HA- and Myc-tagged proteins (input). (**B**) Prion propagation assay of the dominant-negative ΔN-p73β isoform in yeast. Wild-type FL-p53α expression was placed under the control of the constitutive *GPD* promoter whereas the expression of ΔN-p73β isoform was placed under the control of the glucose-repressible *GAL* promoter which is switched ON by galactose and switched OFF by glucose.

As shown in this study, the capacity of mutants and isoforms of p53 to disrupt the transcriptional activity of p53 canonical isoform requires a physical interaction with p53-WT through their tetramerization domain, which indicates that their dominant-negative property is likely due to tetramer poisoning (Figure 4). We thus investigated if the same mechanism was responsible for the dominant-negative effect of p53-R175H over p63 and p73 transactive isoforms TA-p63γ and TA-p73γ using co-immunoprecipitation. We found that TA-p63γ and TA-p73γ strongly interacted with p53-R175H but only weakly with FL-p53α-WT (Figure 6A), as previously described [36, 76, 77]. p53-R175H interaction with TA-p63γ and TA-p73γ was markedly impaired but not completely abrogated by L344P mutation suggesting that p53-R175H/TA-p63γ and p53-R175H/TA-p73γ interactions did not solely depend on the tetramerization ability of p53-R175H (Figure 6A). Our results indicate that p53-R175H dominant-negative effect over TA-p63γ and TA-p73γ involves only partially the mutant tetramerization capacity, which suggests that a mechanism other than tetramer poisoning may be involved in the dominant-negative interplay between p53 and p63 and p73.

### Dominant-negative effect of ΔN-p73α and ΔN-p73β over p53, p63 and p73 transactive isoforms

We then assessed the dominant-negative potential of the inactive ΔN-p73α and ΔN-p73β. Our results that are summarized in Table 2 showed that loss-of-function isoforms of p73 interfere with active isoforms of p53, p63 and p73 (Supplementary Figures S2D and S3). We finally determined that the dominant-negative effect exerted by ΔN-p73β over FL-p53α-WT did not rely on a prion-like mechanism in yeast because it was incapable of self-propagating in FASAY-RGC (Figure 6B) and FASAY-p21 (Supplementary Figure S2E) using the same glucose-repressible shut-off system used in Figure 4A and Supplementary Figure S2A. Therefore our results indicate that the dominant-negative effect is widely spread across the p53, p63 and p73 family of genes.

**Table 2:** Dominant-negative effect of ΔN-p73α and ΔN-p73β over all transactive isoforms of the p53 family (Supplementary Figures S2D and S3)

	ΔN-p73α	ΔN-p73β
**FL-p53α**	+	++
**Δ40-p53α**	+	++
**TA-p63α**	−	−
**TA-p63β**	−	−
**TA-p63γ**	+	+
**ΔN-p63α**	−	−
**ΔN-p63β**	−	++
**ΔN-p63γ**	−	+++
**TA-p73α**	−	−
**TA-p73β**	−	−
**TA-p73γ**	++	+
**ΔN-p73γ**	−	+

## DISCUSSION

The loss of the tumor suppression function of p53, p63 and p73 is a crucial event of tumorigenesis that is partly due to a dominant-negative effect exerted by loss-of-function mutants and isoforms of these three genes over their active counterparts. However, the underlying mechanisms involved remain poorly defined and no extensive study in a unique system has been performed before this study. In this work, we investigated the dominant-negative effect of p53, p63 and p73 in yeast, a naive and homogeneous eukaryotic system. We first focused on p53 hotspot mutants as they account for 30% of all p53 reported mutations. We found mutants R175H, G245S, R248Q, R249S and R273H to be transcriptionally inactive and dominant-negative over their wild-type counterpart in agreement with previous results [32, 63]. Of note, although mutants of p53 have been classified as “contact mutants” (R248Q, R273H) or “conformational mutants” (R175H, R249S), we found no difference in their ability to interfere with WT p53 transcriptional activity. These data support the idea that such classification may be an oversimplification [78] and thereby suggest the existence of a common mechanism in their trans-dominance over p53. However, loss of transcriptional activity alone is not sufficient to lead to a dominant-negative activity as evidenced by the analysis of β and γ isoforms of p53. Indeed, we found that all β and γ isoforms of p53 tested were transcriptionally inactive which is in good agreement with part of the literature [79]. Their inactivity is either due to an altered TAD and/or an impaired tetramerization domain, but none of these 8 isoforms exhibited a dominant-negative effect. In contrast, Δ133-p53α and Δ160-p53α, which only differ from their β and γ counterparts by an intact tetramerization domain, possess a dominant-negative activity over p53-WT. Our work then demonstrated that the ability of inactive forms of p53 to interact with active forms of p53 is the cornerstone of their dominant-negative effect. The dominant-negative effect also depends on the loss of transcriptional activity of mutants and isoforms on the response element involved. Since hotspot mutants of p53 suffer from a severe loss of transcriptional activity affecting several response elements [32, 80], their dominant-negative effect is likely to affect p53 in most cases. The transcriptional spectrum of Δ133-p53α and Δ160-p53α remains to be determined but we can speculate that their loss of function would be as severe as hotspot mutants of p53 due to their structural defect.

In addition, we showed that dominant-negative R175H, R248Q, R273H, Δ133-p53α and ΔN-p73β are unable to transmit their dominant-negative features to WT-p53 protein through a prion-based mechanism. The p53 prion field is somehow tainted by the shortcut that an amyloid is an infectious prion [81]. Numerous proteins can form amyloid aggregates [82], among which p53 [38, 43, 83], but only few are able to transmit their particular conformation to natively folded proteins, which corresponds to the definition of a prion. Yeast has been used to demonstrate the propagation features of various prion proteins [71, 84–88] and our data challenge the prion potential of p53 dominant-negative mutants. Indeed, using an ON/OFF system to drive p53 mutants and isoform expression in yeast, we showed that p53 dominant-negative mutants are not able to transmit their dominant-negative feature to WT p53 proteins from cell to cell contrary to previous reports [41, 42, 83]. Thus the *in vivo* evidence provided here do not support the recently proposed model of a prion-based dominant-negative effect of p53 mutants.

Hence, the critical condition required for an inactive p53 protein to exert a dominant-negative effect is to retain the ability to physically interact with p53 through its tetramerization domain. Therefore, preventing the formation of hetero-tetramers that combine active and inactive forms of p53 represents an attractive and relevant therapeutic target to reactivate p53 activity in tumors containing both transcriptionally inactive hotspot mutants of p53 and WT p53. However, specifically targeting p53 hetero-tetramers without affecting active p53 homo-tetramers may be particularly tricky. Promising work tackled this issue through the design of a chimeric p53 harboring the coiled-coil domain of Bcr as an alternate tetramerization domain [89].

Importantly, the dominant-negative interference is not limited to p53 and also involves p63 and p73 isoforms. In this work we provided evidence for ΔN-p63 and ΔN-p73 being transcriptionally active although they have been depicted as loss-of-function dominant-negative regulators [15]. All ΔN-p63 isoforms were indeed found to be transcriptionally active indicating that the alternate TA domain created by splicing does not abolish their transactive potential but rather expands it. We found that TA-p63α has a low transcriptional activity on *RGC* response element but the activity of this isoform was previously shown to depend on the response element used [75]. In contrast, ΔN-p73α and ΔN-p73β were inactive in FASAY-RGC but weakly active in FASAY-p21, which is in good agreement with the data reported by Monti et al. [54]. Interestingly, we found that ΔN-p73α and ΔN-p73β were only barely functional on p21-RE and not functional on RGC RE, whereas ΔN-p73γ was functional on both response elements, suggesting that the C-terminal end of these isoforms may be important for RE recognition and/or activation.

The alternate TA domain of ΔN-p73 isoforms thus does not prevent transcription activation capacities whereas the 3′ splicing producing these isoforms leads to major modification of the isoforms transcriptional function. Several reports demonstrated that the alternate N-terminal extremity of these isoforms can also serve as a transcription activation domain. Indeed ΔN isoforms of p63 and p73 are potent transcriptional regulators whose targets partially diverge from that of their TA counterparts [52–55]. Among p63 and p73 isoforms, only ΔN-p73α and ΔN-p73β were dominant-negative in our system. They were indeed able to interfere with p53, p63 and p73 transcriptional activity in FASAY-RGC. However, previous works have shown that p73 isoforms were able to interact with isoforms of p63 and p73 but not with p53 [90, 91]. This suggests that the dominant-negative capacity of ΔN-p73α and ΔN-p73β may be due to the formation of inactive hetero-tetramers with functional isoforms of p63 and p73 and/or another mechanism that does not involve a direct interaction such as competition for binding sites as suggested by Grob et al. [92].

Our results regarding the transcriptional activity and dominant-negative potential of mutants and isoforms of p53, p63 and p73 have been summarized and compared to data from the literature (Supplementary Table S2). To our knowledge, they mostly agree with previous data obtained by different groups using various models. The few observed discrepancies may arise from the different types of reporter genes used ([53–55, 92, 93], Supplementary Table S2), the likely presence of other isoforms ([56], Supplementary Table S2) or the use of temperature-sensitive mutants ([32, 80], Supplementary Table S2).

Finally, our findings showed that R175H ability to interact with p63 and p73 was increased compared to p53-WT, as previously described [94]. Previous evidence indicate that the unfolded core-domain of p53 mutants is sufficient to allow them to interact with p63/p73 isoforms [34, 77, 94]. However, we found that an intact tetramerization ability of R175H significantly increases its interaction with p63/p73 suggesting that tetramers of R175H interfere with p63 and p73. As each mutation of p53 may lead to a different conformational change, the interaction spectrum of p53 mutants needs to be further characterized. Such mechanism could explain enhanced resistance to chemotherapies observed in cancers harboring p53 mutations [94] and therefore may lead to the use of new biological markers and targets in cancer treatments. However, how these interactions lead to the inactivation of functional isoforms remains to be determined although it could be related to the formation of protein aggregates that sequester p53, p63 and p73 as reported by Xu et al. [38]. Several drugs are currently evaluated for their capacity to reactivate p53 mutants by refolding their destabilized core domain or by disrupting the interaction between p53 mutants and p63/p73 [95].

## MATERIALS AND METHODS

### Yeast strains

*Saccharomyces cerevisiae* p53-reporter strains FASAY-RGC (yIG397) [30] and FASAY-p21 (YPH-p21) [62] were kind gifts of JM. Flaman. Yeast cells were transformed using lithium acetate method [96].

### Creation of mutants and isoforms

p53 mutants were created by site-directed mutagenesis (QuickChange Lightning, Agilent technologies, Santa Clara, California, USA) according to the manufacturer instructions. p53, p63 and p73 isoforms were generated by PCR (Supplementary Table S1).

### Construction of the expression vectors

Plasmids harboring FL-p53^WT/P72R^ (P04637-1) and FL-p53^R72P/R175H^ were gifts from L. Maillet (UMR 892, Nantes, France), Δ133-p53α (P04637-7), Δ133-p53β (P04637-8) and Δ133-p53γ (P04637-9) were gifts from P. Roux (CRBM, Montpellier, France), and TA-p73α (O15350–1), TA-p73β (O15350–2), TA-p73γ (O15350–3), ΔN-p73α (O15350–8), ΔN-p73β (O15350–9) and TA-p63α (Q9H3D4-1) were gifts from B. Vojtesek (Masaryk Memorial Cancer Institute, Brno, Czech Republic). Cloning restriction sites BamHI/EcoRI were added by PCR respectively to the N-term and C-term for p53 mutants and isoforms (Supplementary Table S1). Restriction sites BamHI/ClaI were used for p63 and p73 isoforms. HA- or Myc-Tag was added by PCR (Supplementary Table S1) in N-term (p63 and p73 isoforms) or C-term (p53 mutants and isoforms).

Yeast expression vectors pRS413 (*HIS3*), pRS414 (*TRP1*), and pRS415 (*LEU2*) [97, 98] were used to express the cDNA of human p53/p63/p73 family members under the control of *CYC1*, *ADH1*, *TEF1*, *GPD* or *GAL1* promoters.

### Transactivation assay

p53, p63 and p73 were expressed in FASAY strains from a pRS413 vector under the control of the indicated promoter.

### Transdominance assay

Functional members of p53 family were co-expressed at their minimal transactivation level from pRS413 vector under the control of the indicated promoter, together with a mutant or isoform of p53 family from p414-*GPD* alone or together with p415-*GPD*.

### Cell lysis and Western blotting

5 mL of 0.6 O.D._600_ of exponentially growing cultures were harvested by centrifugation and cell pellets were boiled for 5 min in 250 μL of ACB (25 mM Tris-HCl (pH 6.8), 10% glycerol, 5% 2-mercaptoethanol, 2% SDS, 8 M urea). Proteins were immunostained using the indicated primary antibodies (Rat anti-HA 3F10, Roche, Basel, Switzerland; mouse anti-Myc 9E10 631206, Clontech, Mountain View, California, USA; mouse anti-p73 5B1288, Novus Biologicals, Littleton, Colorado, USA; mouse anti-GAPDH ab125247, Abcam, Cambridge, UK; rabbit anti-Arp5 ab12099, Abcam) and HRP-conjugated secondary antibodies (rabbit anti-mouse, Dako, Glostrup, Denmark; swine anti-rabbit, Dako) as instructed by manufacturers.

### Analysis of the propagation of the dominant-negative phenotype in yeast

FASAY-RGC cells were transformed with pRS413-*ADH*-FL-p53α-WT-HA and pRS416-*GAL1*-FL-p53α-R175H-Myc, -Δ133-p53α-WT-Myc or -ΔN-p73β-Myc and plated on selective medium containing 2% glucose to repress *GAL1*-mediated expression. Transformed yeasts were then streaked onto glucose-free selective medium containing 2% galactose and 2% raffinose to allow *GAL1*-mediated expression. Co-expression of functional FL-p53α-WT-HA with dominant-negative FL-p53α-R175H-Myc, Δ133-p53α-WT-Myc or ΔN-p73β-Myc leads to the formation of red colonies. Yeast colonies were then streaked from glucose-free to 2% glucose-containing medium to repress *GAL1*-mediated expression.

### Co-immunoprecipitation

100 mL of 0.6 O.D._600_ of exponentially growing cultures were suspended in 500 μL of IP lysis buffer (Pierce Biotechnology/Thermofisher, Waltham, Massachusetts, USA) containing 1X cOmplete (Roche) and 1 mM Pefabloc (Sigma-Aldrich, St. Louis, Missouri, USA). After addition of 500 μL of glass beads, samples were broken as previously described [72] and then disrupted on a glass-bead beater (MM400 Retsch, Haan, Germany) at 25 Hz at 4°C. 100 μL of 20% protein G-Sepharose fast flow (Sigma-Aldrich) were washed in wash buffer (PBS 1×/0.2% Igepal) then suspended in 500 μL washing buffer. Protein G-Sepharose were mixed with 1.5 μg of rat anti-HA 3F10 for 2h at 4°C. Beads were washed 3 times with washing buffer and incubated with 1 mg of total protein overnight at 4°C. Beads were then washed 5 times with washing buffer and boiled with 65 μL of ACB. Samples were analyzed by western blotting using the indicated primary (Rabbit anti-HA 631207, Clontech; mouse anti-Myc 9E10, Clontech; mouse anti-GAPDH ab125247, Abcam) and secondary antibodies (swine anti-rabbit, Dako; anti-mouse Veriblot, Abcam).

## SUPPLEMENTARY MATERIALS


