# Supplementary material for: The dominant-negative interplay between p53, p63 and p73: A family affair
Source: Oncotarget. 2016 Aug 31;7(43):69549–64. doi: 10.18632/oncotarget.11774 (PMC5342497; doi:10.18632/oncotarget.11774)
Supplement: Supplementary file 1 [file oncotarget-07-69549-s001.pdf]

The dominant-negative interplay between p53, p63 and p73: A family affair

Supplementary Materials

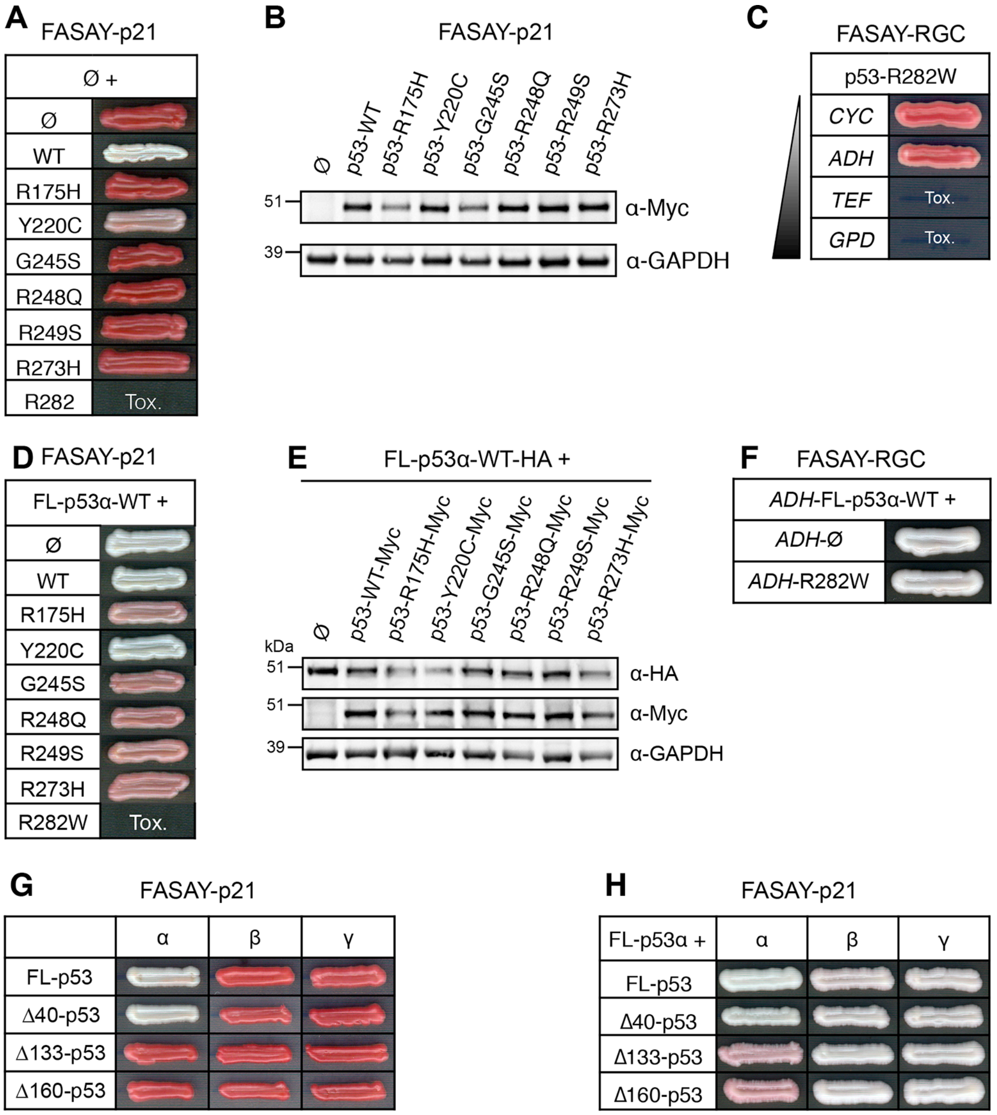

Supplementary Figure S1: (A) Transcriptional activity of hotspot p53 mutants expressed in FASAY-p21 yeast strain. All hotspot mutants were expressed under the control of the strong *GPD* promoter. (**B**) Expression level of Myc-tagged p53 mutants in FASAY-p21 strain was analyzed by western blotting. GAPDH was used as a loading control. (**C**) Mutant p53-R282W was expressed in FASAY-RGC strain under the control of promoters of increasing strength (*CYC*<*ADH*<*TEF*<*GPD*). Tox indicates the absence of cell growth due to an excessive level of expression of the isoform. (**D**) Transdominance assay of hotspot mutants of p53 over FL-p53α-WT in FASAY-p21 yeast strain. Mutants were expressed under the control of the strong *GPD* promoter and FL-p53α-WT was expressed under the control of the moderate *ADH* promoter. (**E**) Western blot analysis of the expression level of FL-p53α-WT-HA and mutant FL-p53α-Myc in FASAY-p21 strain using anti-HA and anti-Myc antibodies. GAPDH was used as a loading control. (**F**) Transdominance assay of R282W hotspot p53 mutant over FL-p53α-WT in FASAY-RGC. R282W was expressed under the control of the strong *ADH* promoter. (**G**) Transcriptional activity of p53 family isoforms in FASAY-p21. (**H**) Dominant-negative effect of FL-, Δ40-, Δ133- and Δ160-p53 isoforms over FL-p53α-WT in FASAY-p21. Isoforms of p53 were expressed under the control of the strong *GPD* promoter and FL-p53α-WT-HA was expressed under the control of the moderate *ADH* promoter.

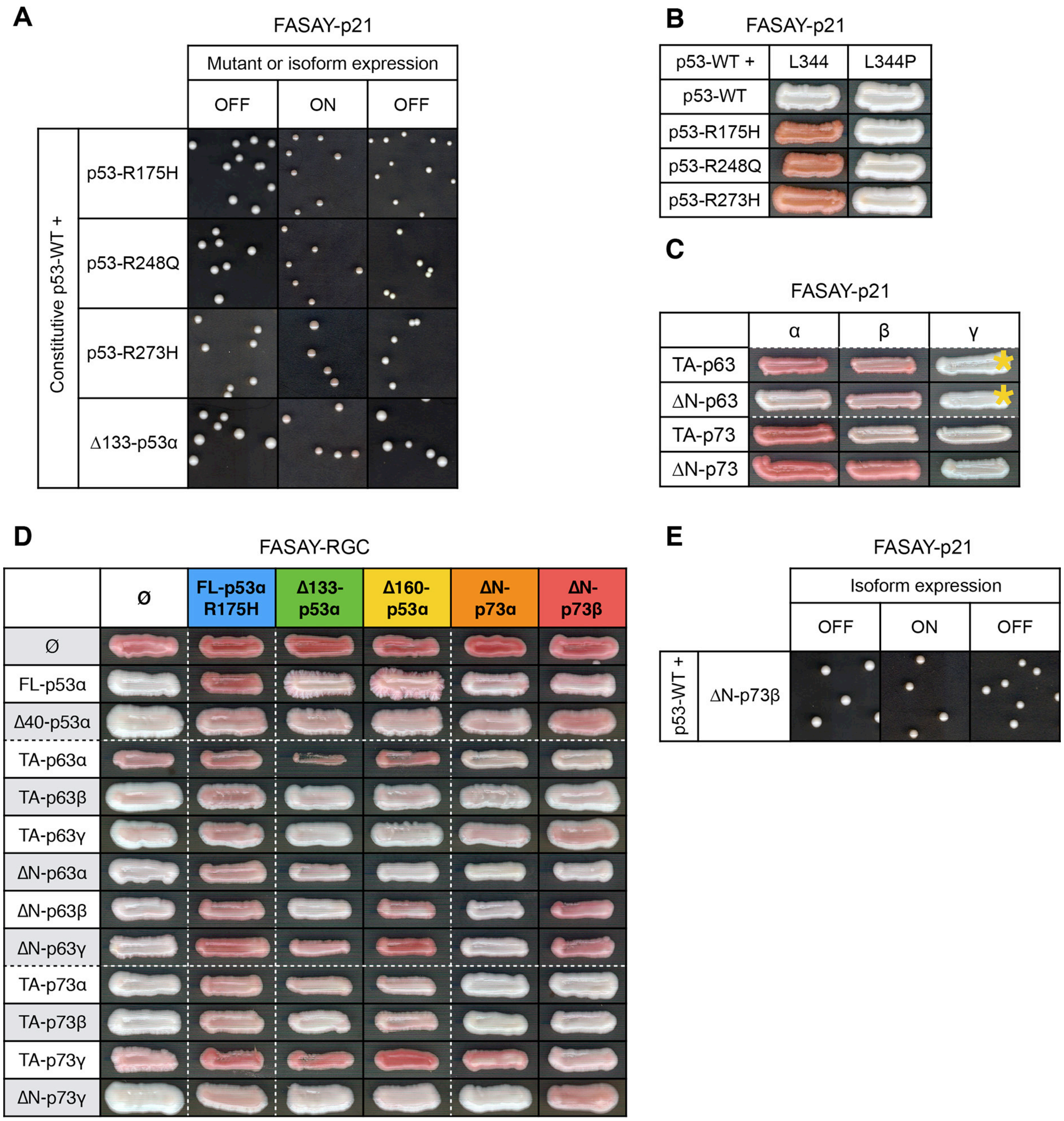

Supplementary Figure S2: (**A**) Prion propagation assay of the dominant-negative R175H, R248Q and R273H mutants and Δ133-p53α isoform in FASAY-p21 yeast strain. Wild-type FL-p53α expression was placed under the control of the *GPD* constitutive promoter whereas the expression of R175H, R248Q and R273H mutants and Δ133-p53α isoform were put under the control of the glucose-repressible *GAL* promoter which is switched ON by galactose and OFF by glucose. (**B**) Impact of the tetramerization disruptive L344P mutation on dominant-negative mutants and on Δ133-p53α isoform of p53 in FASAY-p21 strain. R175H, R248Q and R273H mutants and Δ133-p53α isoform, and the double mutants R175H-L344P, R248Q-L344P and-R273H-L344P and the Δ133-p53α-L344P mutant isoform placed under the control of the strong *GPD* promoter were co-expressed with FL-p53α-WT placed under the control of the moderate *ADH* promoter. (**C**) Transcriptional activity of the 6 main isoforms of p73 expressed in FASAY-p21 strain under the control of the strong *GPD* promoter. Yellow asterisks indicate isoforms that were expressed under the control of the moderate *ADH* promoter because of their toxicity when expressed from the *GPD* promoter. (**D**) Dominant-negative effect of mutant FL-p53α-R175H and isoforms Δ133-p53α, Δ160-p53α, ΔN-p73α and ΔN-p73β over all transactive isoforms of the p53 family. Transactive isoforms were co-expressed at their minimal transactivity level with 1 (for weak and moderate isoforms, see Figure 5E) or 2 (for strong isoforms, see Figure 5E) together with plasmids expressing p53-R175H, Δ133-p53α, Δ160-p53α, ΔN-p73α and ΔN-p73β under the control of the strong *GPD* promoter. FL-p53α-R175H exerted a strong effect over FL-p53α, ΔN-p63γ and TA-p73γ as well as a mild effect over all the other isoforms tested. Δ133-p53α induced a mild effect over FL-p53α, Δ40-p53α, ΔN-p63β, TA-p73α and TA-p73γ. Δ160-p53α exerted a strong effect over FL-p53α, ΔN-p63β and TA-p73γ as well as a mild effect over Δ40-p53α, ΔN-p63β, TA-p73α and TA-p73γ. ΔN-p73α exerted a mild dominant-negative effect over FL-p53α, Δ40-p53α and TA-p73γ. ΔN-p73γ showed a broader spectrum of dominance as it exerted a strong effect over Δ40-p53α, ΔN-p63β and ΔN-p63β as well as a mild effect over FL-p53α, TA-p63γ, TA-p73γ and ΔN-p73γ. (**E**) Prion propagation assay of the dominant-negative ΔN-p73β isoform in FASAY-p21 yeast strain performed in the conditions described in panel (A).

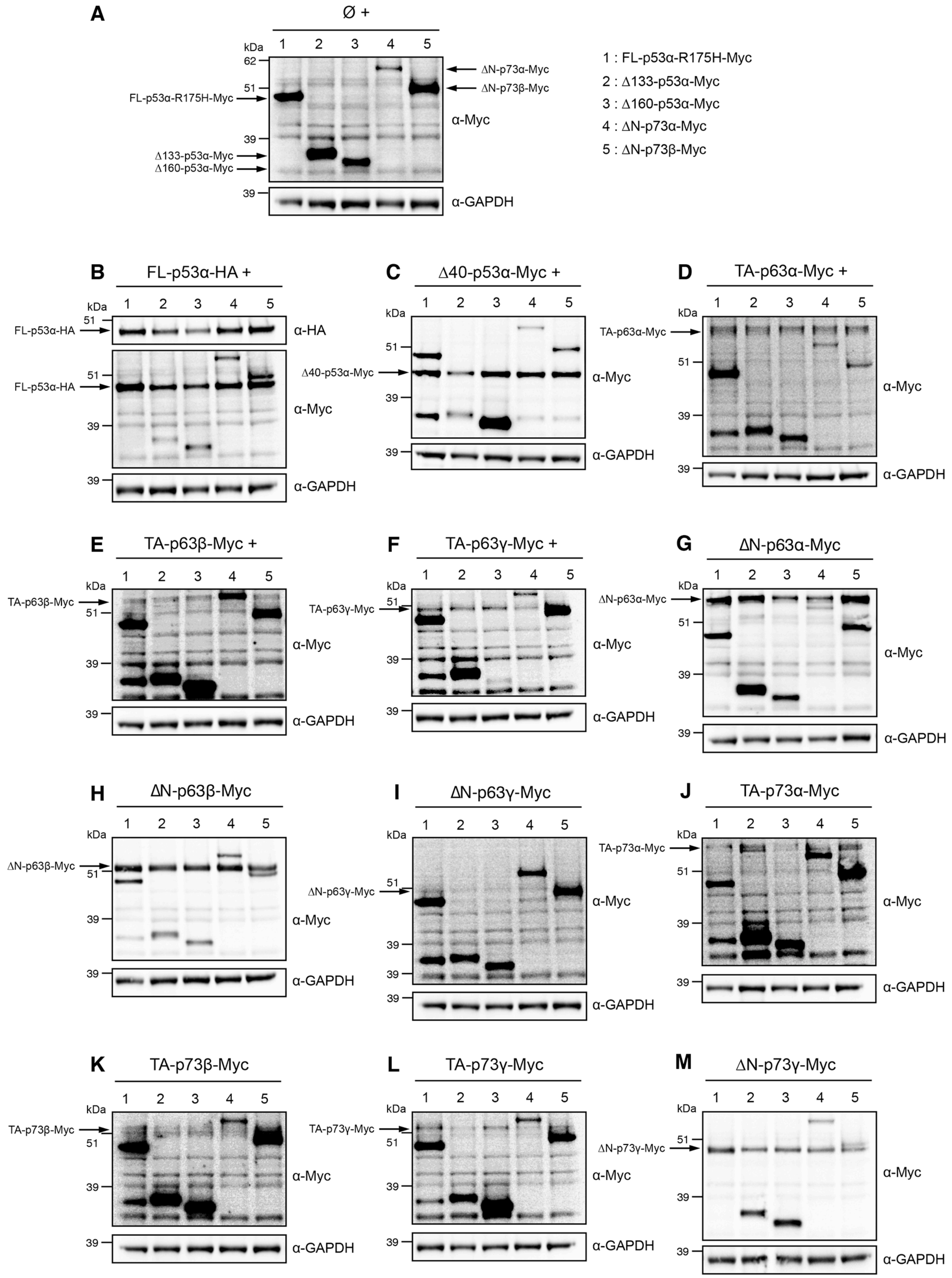

Supplementary Figure S3: Expression level of dominant-negative p53 mutants and p53 and p73 isoforms in FASAY-RGC strain was analysed by western blotting. Myc-tagged p53-R175H (lane 1), Δ133-p53α (lane 2), Δ160-p53α (lane 3), ΔN-p73α (lane 4) and ΔN-p73β (lane 5) were visualised using anti-Myc antibody in panels A to M. Panel (**A**) is a control panel representing dominant-negative proteins only. Expression of transactive wild-type FL-p53α (**B**) was visualised using anti-HA antibody. Δ40-p53α isoform (**C**), p63 isoforms TA-p63α (**D**), TA-p63β (**E**), TA-p63γ (**F**), ΔN-p63α (**G**), ΔN-p63β (**H**) and ΔN-p63γ (**I**) expression level was analyzed using anti-Myc antibody. p73 isoforms TA-p73α (**J**), TA-p73β (**K**), TA-p73γ (**L**) and ΔN-p73γ (**M**) were detected using anti-p73 antibody after Myc detection of the transactive proteins. GAPDH was used as a loading control.

Supplementary Table S1: Primers used to construct mutants and isoforms of p53, p63 and p73.
See Supplementary_Table_S1

Supplementary Table S2: Summary of the transcriptional activity and dominant-negative potential data from the present study

Isoform or mutant	Transcriptional activity		Dominant-negative effect	
	Yes	No	Yes	No
FL-p53α	✦			✦
FL-p53β	17	✦[15 ; 16 ; 17]		✦[16 ; 17]
FL-p53γ		✦[16]		✦[16]
Δ40-p53α	✦* [17]	9	9 ; 17	✦
Δ40-p53β		✦		✦
Δ40-p53γ		✦		✦
Δ133-p53α		✦[17]	✦[15 ; 17]	
Δ133-p53β		✦[16]		✦[16]
Δ133-p53γ		✦[16]		✦[16]
Δ160-p53α		✦	✦	
Δ160-p53β		✦		✦
Δ160-p53γ		✦		✦
TA-p63α	✦[7 ; 10 ; 19]	4		✦
TA-p63β	✦[10 ; 19]			✦
TA-p63γ	✦[4 ; 10]			✦
ΔN-p63α	✦[7 ; 19]	4 ; 10	4	✦
ΔN-p63β	✦[10 ;19]			✦
ΔN-p63γ	✦[10]	4	4	✦
TA-p73α	✦[19]			✦
TA-p73β	✦[19]			✦
TA-p73γ	✦			✦
ΔN-p73α	✦*** [14]	✦** [8 ; 19]	✦** [8 ; 11]	✦***
ΔN-p73β	✦*** [13]	✦** [19]	✦**	✦***
ΔN-p73γ	✦[13]			✦
p53-R175H		✦[1 ; 5 ; 12 ; 18]	✦[6 ; 18]	
p53-Y220C	✦* [5]	12 ; 18	18	✦[6]
p53-G245S		✦[12 ; 18]	✦[3 ; 6 ; 18]	
p53-R248Q		✦[1 ; 12 ; 18]	✦[3 ; 6 ; 18]	
p53-R249S		✦[1 ; 5 ; 12]	✦[3 ; 6]	
p53-R273H		✦[1 ; 5 ; 12 ; 18]	✦[3 ; 6 ; 18]	
p53-R282W		✦[12 ; 18]	✦[3 ; 18]	
p53-L344P		✦[2 ; 12 ; 18]	18	✦

✦Results from present study.
*Partial transcriptional activity.
**On RGC-RE only.
***On p21-RE only.

REFERENCES

1. Ishioka C, Frebourg T, Yan YX, Vidal M, Friend SH, Schmidt S, Iggo R. Screening patients for heterozygous p53 mutations using a functional assay in yeast. Nat Genet. 1993; 5:124–9.
2. Ishioka C, Englert C, Winge P, Yan YX, Engelstein M, Friend SH. Mutational analysis of the carboxy-terminal portion of p53 using both yeast and mammalian cell assays *in vivo*. Oncogene. 1995; 10:1485–92.
3. Brachmann RK, Vidal M, Boeke JD. Dominant-negative p53 mutations selected in yeast hit cancer hot spots. Proc Natl Acad Sci. 1996; 93:4091–5.
4. Yang A, Kaghad M, Wang Y, Gillett E, Fleming MD, Dötsch V, Andrews NC, Caput D, McKeon F. p63, a p53 homolog at 3q27–29, encodes multiple products with transactivating, death-inducing, and dominant-negative activities. Mol Cell. 1998; 2:305–16.
5. Como CJD, Prives C. Human tumor-derived p53 proteins exhibit binding site selectivity and temperature sensitivity for transactivation in a yeast-based assay. Oncogene. 1998; 16:2527–39.
6. Marutani M, Tonoki H, Tada M, Takahashi M, Kashiwazaki H, Hida Y, Hamada J, Asaka M, Moriuchi T. Dominant-negative mutations of the tumor suppressor p53 relating to early onset of glioblastoma multiforme. Cancer Res. 1999; 59:4765–9.
7. Dohn M, Zhang S, Chen X. p63α and ΔNp63α can induce cell cycle arrest and apoptosis and differentially regulate p53 target genes. Oncogene. 2001; 20:3193–205.
8. Grob TJ, Novak U, Maisse C, Barcaroli D, Lüthi AU, Pirnia F, Hügli B, Graber HU, De Laurenzi V, Fey MF, others. Human delta Np73 regulates a dominant negative feedback loop for TAp73 and p53. Cell Death Differ. 2001; 8:1213–23.
9. Courtois S, Verhaegh G, North S, Luciani MG, Lassus P, Hibner U, Oren M, Hainaut P. DeltaN-p53, a natural isoform of p53 lacking the first transactivation domain, counteracts growth suppression by wild-type p53. Oncogene. 2002; 21:6722–8.
10. Ghioni P, Bolognese F, Duijf PHG, van Bokhoven H, Mantovani R, Guerrini L. Complex Transcriptional Effects of p63 Isoforms: Identification of Novel Activation and Repression Domains. Mol Cell Biol. 2002; 22:8659–68.
11. Zaika AI, Slade N, Erster SH, Sansome C, Joseph TW, Pearl M, Chalas E, Moll UM. ΔNp73, A Dominant-Negative Inhibitor of Wild-type p53 and TAp73, Is Up-regulated in Human Tumors. J Exp Med. 2002; 196: 765–80.
12. Kato S, Han S-Y, Liu W, Otsuka K, Shibata H, Kanamaru R, Ishioka C. Understanding the function–structure and function–mutation relationships of p53 tumor suppressor protein by high-resolution missense mutation analysis. Proc Natl Acad Sci. 2003; 100:8424–9.
13. Liu G, Nozell S, Xiao H, Chen X. ΔNp73 Is Active in Transactivation and Growth Suppression. Mol Cell Biol. 2004; 24:487–501.
14. Tanaka Y, Kameoka M, Itaya A, Ota K, Yoshihara K. Regulation of HSF1-responsive gene expression by N-terminal truncated form of p73α. Biochem Biophys Res Commun. 2004; 317:865–72.
15. Bourdon JC. p53 isoforms can regulate p53 transcriptional activity. Genes Dev. 2005; 19:2122–37.
16. Graupner V, Schulze-Osthoff K, Essmann F, Jänicke RU. Functional characterization of p53β and p53γ, two isoforms of the tumor suppressor p53. Cell Cycle. 2009; 8:1238–48.
17. Khoury MP, Bourdon JC. p53 Isoforms: An Intracellular Microprocessor? Genes Cancer. 2011; 2:453–65. doi: 10.1177/1947601911408893.
18. Monti P, Perfumo C, Bisio A, Ciribilli Y, Menichini P, Russo D, Umbach DM, Resnick MA, Inga A, Fronza G. Dominant-negative features of mutant p53 in germline carriers have limited impact on cancer outcomes. Mol Cancer Res. 2011; 9:271–9.
19. Monti P, Ciribilli Y, Bisio A, Foggetti G, Raimondi I, Campomenosi P, Menichini P, Fronza G, Inga A. ΔN-P63α and TA-P63α exhibit intrinsic differences in transactivation specificities that depend on distinct features of DNA target sites. Oncotarget. 2014; 5:2116. doi: 10.18632/oncotarget.1845.
